# Modeling Nosocomial Infections of Methicillin-Resistant *Staphylococcus aureus* with Environment Contamination^*^

**DOI:** 10.1038/s41598-017-00261-1

**Published:** 2017-04-03

**Authors:** Lei Wang, Shigui Ruan

**Affiliations:** Department of Mathematics, University of Miami, Coral Gables, FL 33146 USA

## Abstract

In this work, we investigate the role of environmental contamination on the clinical epidemiology of antibiotic-resistant bacteria in hospitals. Methicillin-resistant *Staphylococcus aureus* (MRSA) is a bacterium that causes infections in different parts of the body. It is tougher to treat than most strains of *Staphylococcus aureus* or staph, because it is resistant to some commonly used antibiotics. Both deterministic and stochastic models are constructed to describe the transmission characteristics of MRSA in hospital setting. The deterministic epidemic model includes five compartments: colonized and uncolonized patients, contaminated and uncontaminated health care workers (HCWs), and bacterial load in environment. The basic reproduction number *R*_0_ is calculated, and its numerical and sensitivity analysis has been performed to study the asymptotic behavior of the model, and to help identify factors responsible for observed patterns of infections. A stochastic epidemic model with stochastic simulations is also presented to supply a comprehensive analysis of its behavior. Data collected from Beijing Tongren Hospital will be used in the numerical simulations of our model. The results can be used to provide theoretical guidance for designing efficient control measures, such as increasing the hand hygiene compliance of HCWs and disinfection rate of environment, and decreasing the transmission rate between environment and patients and HCWs.

## Introduction

The emergence and spread of antimicrobial-resistant bacteria (ARB) is one of the most serious public health threats. Bacteria such as vancomycin-resistant enterococci (VRE) and glycopeptide-intermediate sensitive *Staphylococcus aureus* present hospitals with the prospect of a postantibiotic era, in which few if any therapeutic antimicrobial agents remain effective (Weinstein *et al.* ^1^). Compared to infections caused by susceptible strains, infections caused by antibiotic-resistant organisms are more likely to prolong hospitalization, to increase the risk of death, and to require treatment with more toxic or more expensive antibiotics (Flaherty and Weinstein^2^). Patients admitted to healthcare institutions are the main reservoirs of ARB. It is estimated that 5–10% of patients develop an infection directly related to their hospitalization, resulting in over 90,000 deaths per year in the US. Infections that are acquired in hospitals, and are favored by a hospital environment, referred to by the technical term ‘nosocomial’ have been a big threat to the public health. This situation is even more severe in China. A high percentage of hospital-acquired infections are caused by highly resistant bacteria such as methicillin-resistant *Staphylococcus aureus* (MRSA) and vancomycin-resistant enterococci. In particular, methicillin-resistant *Staphylococcus aureus* (MRSA) is associated with considerable morbidity and mortality among inpatients (Cosgrove *et al.* ^3, 4^) and accounts for 35–80% of total staphylococcal infection in China (Wang *et al.* ^5^). Patients colonized with MRSA are more likely to develop infection (Niven *et al.* ^6^, Reilly *et al.* ^7^).

Considerable quantitative research has been dedicated into the study of infection control strategies. In order to determine the numerous interrelated factors that contribute to the spread of various infectious diseases, many mathematical models have proposed to study the epidemiology of infectious diseases. This is especially true for the description of the transmission dynamics of infectious diseases ranging from measles and pertussis to gonorrhea and in the prediction of the effects of public health interventions such as treatment and vaccination on these dynamics (Anderson and May^8^, Keeling and Rohani^9^). In the last two decades, mathematical modeling has provided a very useful means to study the transmission dynamics of nosocomial pathogens in hospitals, including investigations of patient and health care worker (HCW) contact patterns, and HCW- and patient-mediated transmission, we refer to Austin and Anderson^10^, Bergstrom *et al.* ^11^, Bonten *et al.* ^12^, Chamchod and Ruan^13^, Cooper *et al.* ^14^, D’Agata *et al.* ^15–17^, Grundmann and Hellriegel^18^, Kribs-Zaleta *et al.* ^19^, Plipat *et al.* ^20^, Smith *et al.* ^21^, Webb *et al.* ^22, 23^, Lipsitch *et al.* ^24^, and the references cited therein.

Pathogens such as MRSA are capable of surviving for days, weeks or even months on environmental surfaces in healthcare facilities computer keyboards to equipment packaging to patients gowns (Dancer^25^). Much evidence has been proposed to show that environmental contamination is an important factor in the transmission of MRSA (Boyce *et al.* ^26^). Environmental contamination may contribute to transmission of pathogens when health care workers contaminate their hands or gloves by touching contaminated surfaces, or when patients come into direct contact with contaminated surfaces. Transmission of MRSA from environmental surfaces to gloves or hands of HCWs has been documented by several investigators (Boyce^27^). Though there are some studies in modeling the impact of environmental contaminations in nosocomial infections (see, for example, McBryde and McElwain^28^, Hall *et al.* ^29^, Wang^30^, Wang *et al.* ^31^, Browne and Webb^32^), little is known about the role of environmental infection in the transmission dynamics of MRSA, and this provides the motivation of our research.

To investigate the transmission pattern of nosocomial infection, we first introduce a deterministic compartmental model of the transmission dynamics of MRSA in hospital with patients, HCWs and bacteria in the environment. Numerical and sensitivity analyses will be carried out to analyze the deterministic model, which concentrates on the interactions between environmental infection and patients and HCWs. A stochastic epidemic model and its simulations are also introduced to check the essential features that are not well described in the deterministic model. Data collected from Beijing Tongren Hospital (Wang *et al.* ^33^) will be used in the numerical simulations of our model.

## Methods

We introduce two nosocomial infection models with environment contamination in hospital, one is deterministic and the other is stochastic.

### Deterministic Models

**Figure 1:**
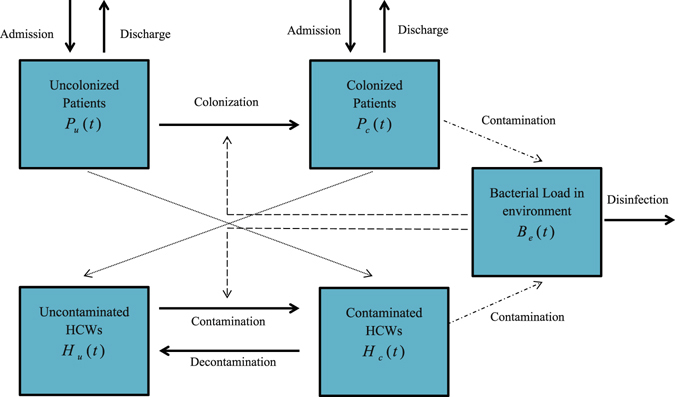
A compartmental model of transmission dynamics of meticillin-resistant *Staphylococcus aureus* among patients and healthcare workers (HCWs) with environmental contamination.

Patients in the hospital unit are classified by compartment as either uncolonized *P*_*u*_(*t*), or colonized *P*_*c*_(*t*); health-care workers are classified as either uncontaminated *H*_*u*_(*t*), or contaminated *H*_*c*_(*t*). The bacterial load in the environment is the compartment *B*_*e*_(*t*). The relation between different compartments inside hospital unit is depicted in the compartmental scheme of Fig. 1. Patients are admitted at a total rate of Λ per day with the fraction of colonized patients *θ*. Since the total number of beds in hospital unit is a fixed number, we assume that the inflow of patients is Λ = *γ*_*u*_ *P*_*u*_ + *γ*_*c*_ *P*_*c*_, based on the assumption of full occupation of the unit, where *γ*_*u*_ and *γ*_*c*_ are discharge rates of uncolonized patients and colonized patients per day from hospital, respectively. Hence, the total number of patients in the unit remains constant at *N*_*p*_. Note that the total number of HCWs is also assumed to be a constant, *N*_*h*_. It is assumed that there is no cross-infection between patients, so that patients can only be colonized with antibiotic-resistant bacteria by contacting contaminated health-care workers *α*_*p*_ *β*_*p*_(1 − *η*)*P*_*u*_(*t*)*H*_*c*_(*t*) or the contaminated environment *k*_*p*_ *P*_*u*_(*t*)*B*_*e*_(*t*), where *α*_*p*_ is the contact rate, *β*_*p*_ is the probability of colonization per contact, *η* is the compliance rate with the hand hygiene, and *k*_*p*_ is the colonization rate from the environment. Health-care workers can be contaminated with antibiotic-resistant bacteria by contacting colonized patients *α*_*p*_ *β*_*h*_(1 − *η*)*P*_*c*_(*t*)*H*_*c*_(*t*) and the contaminated environment *k*_*h*_ *H*_*u*_(*t*)*B*_*e*_(*t*), where *β*_*h*_ is the probability of contamination per contact, and *k*_*h*_ is the contamination rate from the environment. *μ*_*c*_ is the decontamination rate for the HCWs, *ν*_*p*_ and *ν*_*h*_ are the rate that colonized patients and contaminated HCWs contaminate the environment, respectively, and *γ*_*b*_ is the cleaning/disinfection rate of the environment. Details for parameters in this model can be found in Table 1.

**Table 1:** Baseline parameters values and estimates for the transmission MRSA in the Emergency Ward (EW) in Beijing Tongren Hospital (Wang *et al.* ^33^). The unit of time is one day.

Parameter	Symbol	Parameter Estimate	Source
Proportion of colonized patients			
Admitted in hospital (1/day)	*θ*	0.067	33
Number of patients	*N*_*p*_	23	33
Number of HCWs	*N*_*h*_	23	33
Contact rate (1/day)	*α*_*p*_	0.0435	33
Probability of colonization (1/day)			
By colonized patients	*β*_*p*_	0.72	33
By contaminated HCWs	*β*_*h*_	0.20	estimated
Discharge rate (1/day)			
Uncolonized patients	*γ*_*u*_	0.067	33
Colonized patients	*γ*_*c*_	0.046	33
Cleaning / disinfection (1/day)			
rate of environment	*γ*_*b*_	0.7	31
Colonization rate from environment (CFUs/day)			
Of uncolonized patients	*k*_*p*_	0.000004	31
Of uncontaminated HCWs	*k*_*h*_	0.00001	31
Hand hygiene compliance of HCWs	*η*	0.4	33
Decontamination rate of HCWs (1/day)	*μ*_*c*_	24	33
Contamination rate to environment (CFUs/day)			
By colonized patients	*ν*_*p*_	235	estimated
By contaminated HCWs	*ν*_*h*_	235	estimated

The equations of the basic model are

1 $$\left\{\begin{array}{lll}\frac{d{P}_{u}(t)}{dt} & = & (1-\theta )[{\gamma }_{u}{P}_{u}(t)+{\gamma }_{c}{P}_{c}(t)]-{\alpha }_{p}{\beta }_{p}(1-\eta ){P}_{u}(t){H}_{c}(t)\\  &  & -\,{k}_{p}{P}_{u}(t){B}_{e}(t)-{\gamma }_{u}{P}_{u}(t)\\ \frac{d{P}_{c}(t)}{dt} & = & \theta [{\gamma }_{u}{P}_{u}(t)+{\gamma }_{c}{P}_{c}(t)]+{\alpha }_{p}{\beta }_{p}(1-\eta ){P}_{u}(t){H}_{c}(t)+{k}_{p}{P}_{u}(t){B}_{e}(t)-{\gamma }_{c}{P}_{c}(t)\\ \frac{d{H}_{u}(t)}{dt} & = & -{\alpha }_{p}{\beta }_{h}(1-\eta ){P}_{c}(t){H}_{u}(t)+{\mu }_{c}{H}_{c}(t)-{k}_{h}{H}_{u}(t){B}_{e}(t)\\ \frac{d{H}_{c}(t)}{dt} & = & {\alpha }_{p}{\beta }_{h}(1-\eta ){P}_{c}(t){H}_{u}(t)-{\mu }_{c}{H}_{c}(t)+{k}_{h}{H}_{u}(t){B}_{e}(t)\\ \frac{d{B}_{e}(t)}{dt} & = & {\nu }_{p}{P}_{c}(t)+{\nu }_{h}{H}_{c}(t)-{\gamma }_{b}{B}_{e}(t)\end{array}\right.,$$

with initial conditions ${P}_{u}(0)={P}_{u}^{0},{P}_{c}(0)={P}_{c}^{0},{H}_{u}(0)={H}_{u}^{0},{H}_{c}(0)={H}_{c}^{0},{B}_{e}(0)={B}_{e}^{0}$ specified at time 0.

We would like to make some comparisons and comments about model (1) and the model in our previous article (Wang *et al.* ^33^). Indeed, data on HCW, volunteers, patients, and environmental contamination were obtained in the original study. The aim of Wang *et al.* ^33^ was to determine to role of volunteers in the prevalence and persistence of MRSA in Beijing Tongren Hospital, so the environmental contamination factor was not included in the model of Wang *et al.* ^33^. Interestingly, the results in ref. 33 indicate that the involvement of volunteers helps to reduce the spread of MRSA in the hospital, since the interaction between volunteers and patients was one-to-one. The purpose of this article is to study the effect of environmental contamination, for the sake of simplicity, the compartments for volunteers are not included in model (1).

### Disease-free steady state and basic reproduction number

We obtain the basic reproduction number *R*_0_ by using the definition notations and technique of Diekmann *et al.* ^34^ and van den Driessche and Watmough^35^. When *θ* = 0, that is no colonized patients are admitted into hospital, the disease-free equilibrium (DFE) is defined to be

$${E}_{0}=({P}_{u},{P}_{c},{H}_{u},{H}_{c},{B}_{e})=({N}_{p},0,{N}_{h},0,0),$$

where *N*_*p*_, *N*_*h*_ are total number of patients and HCWs, respectively. The basic reproduction number is defined as follows (see the details in the Supplementary Material):

2 $$\begin{array}{lll}{R}_{0} & = & \frac{{k}_{p}{\nu }_{p}{N}_{p}}{2\gamma {\gamma }_{b}}+\frac{{k}_{h}{\nu }_{h}{N}_{h}}{2{\mu }_{c}{\gamma }_{b}}\\  &  & +\frac{\sqrt{{({k}_{p}{\nu }_{p}{\mu }_{c}{N}_{p}-{k}_{h}{\nu }_{h}\gamma {N}_{h})}^{2}+4[({\alpha }_{p}{\beta }_{p}(1-\eta ){\gamma }_{b}+{k}_{p}{\nu }_{h})((1-\eta ){\alpha }_{p}{\beta }_{h}{\gamma }_{b}+{k}_{h}{\nu }_{p}){\mu }_{c}\gamma {N}_{h}{N}_{p}]}}{2\gamma {\mu }_{c}{\gamma }_{b}},\end{array}$$

where *γ* = *θγ*_*u*_ + (1 − *θ*)*γ*_*c*_.

When *θ* = 0, following a result of van den Driessche and Watmough^35^, we know that if *R*_0_ < 1, the disease-free steady state (*N*_*p*_, 0, *N*_*h*_, 0, 0) is locally asymptotically stable; if *R*_0_ > 1, the disease-free steady state is unstable.

There is a general limitation on using *R*_0_. If there is an external source that introduces infection into the system, *R*_0_ can be still regarded as a threshold, but it is impossible to use *R*_0_ to get information about how the system will behave (Brauer and van den Driessche^36^).

### Estimation of parameters

Some parameters are adopted from^31, 33^ and some others are estimated using the data obtained in the original study in Beijing Tongren Hospital as reported in ref. 33.

Based on the assumption that the total number of HCWs remains fixed and bed occupancy is 100%, we have *N*_*p*_ = 23 and *N*_*h*_ = 23. The proportion of colonized patients admitted to hospital is *θ* = 0.067. The daily discharge rates of uncolonized patients and colonized patients are *γ*_*u*_ = 0.067 and *γ*_*c*_ = 0.046, respectively. The hand hygiene compliance of HCWs is *η* = 0.4. The decontamination rate of HCWs is *μ*_*c*_ = 24. The probability of colonization from colonized patients to uncontaminated HCWs is *β*_*p*_ = 0.72.

It is assumed that each patient has one contact from one HCW per day, so that the contact rate between patients and HCWs is ${\alpha }_{p}=\frac{1}{{N}_{h}}$. In Wang *et al.* ^33^, it is assumed that a contaminated HCW has the same ability of transmission as a contaminated volunteer, so the probability of colonization from contaminated HCW to uncolonized patient is *β*_*h*_ = 0.20. We assume that Beijing Tongren Hospital maintains the same standard for the clearance of the environment, so that the cleaning/disinfection rate of the environment is *γ*_*b*_ = 0.7 in the EW unit. In a similar way, the colonization rate from the environment to uncolonized patients and uncontaminated HCWs are *k*_*p*_ = 0.000004 and *k*_*h*_ = 0.00001, respectively, in the EW unit. Note that the units of *k*_*p*_ and *k*_*h*_ is CFUs/day. In microbiology, colony-forming unit (CFU) is a measure of viable bacterial or fungal numbers. Unlike direct microscopic counts where all cells, dead and live, are counted, CFU measures viable cells. It is assumed that a colonized patient and a contaminated HCW have the same effect of contamination of the environment; then the contamination rate to the environment of colonized patients *ν*_*p*_ is equal to the contamination rate to environment of contaminated HCWs *ν*_*h*_, and it is half the value of the shedding rate of patients (Wang *et al.* ^31^), which is 235. The units of *ν*_*p*_ and *ν*_*h*_ is also CFUs/day.

We notice that *γ*_*b*_, *k*_*p*_ and *k*_*h*_ are small, *ν*_*p*_ and *ν*_*h*_ are large. This is because *B*_*e*_ is in units that make it come out to be a large number.

### Stochastic Models

Now we consider the stochastic version of the nosocomial infection model with environmental contamination. The inspiration for introducing the stochastic model comes from Wang *et al.* ^33^. It seems that the number of colonized and uncolonized patients fluctuates randomly due to the small number of patients and HCWs. Meanwhile, simulations using the stochastic model appeared to provide a better explanation of the transmission dynamics for small populations.

There are lots differences between stochastic epidemic models and deterministic ones. For instance, the basic reproduction number can be expressed analytically only in deterministic models. On the other hand, when the sample size is very small, the stochastic simulations can describe the variability of the actual data, which numerical simulations from deterministic models cannot do. The traditional way of obtaining a stochastic epidemic model is based on its deterministic epidemic model (Allen [ref. 37, Chp. 3]). Basically, there are three types of stochastic model formulations: discrete time Markov chain (DTMC), continuous time Markov chain (CTMC) and stochastic differential equations (SDE). These models differ in the underlying assumptions regarding the time and the state variables. We will consider a continuous time Markov chain (CTMC) model for our system, that is to say, the time is continuous, but the state is discrete and embedded in ${{\mathbb{R}}}^{5}$. In order to construct a stochastic epidemic model, we need to consider the probability of changes in variables, the infinitesimal mean and variance, and the drift and diffusion terms of stochastic equation.

It is assumed that ${P}_{u}(t)+{P}_{c}(t)={N}_{p},{H}_{u}(t)+{H}_{c}(t)={N}_{h},\forall \,t\ge 0$, so the process is trivariate {*P*_*c*_(*t*), *H*_*c*_(*t*), *B*_*e*_(*t*)} in ${{\mathbb{R}}}^{3}$, with *P*_*u*_(*t*) = *N*_*p*_ − *P*_*c*_(*t*) and *H*_*u*_(*t*) = *N*_*h*_ − *H*_*c*_(*t*). These three variables have a joint probability given by

3 $${p}_{(s,j,k)}(t)={\rm{Prob}}\{{P}_{c}(t)=s,{H}_{c}(t)=j,{B}_{e}(t)=k\},$$

with *s* = 0, …, *N*_*p*_, *j* = 0, …, *N*_*h*_ and *k* ≥ 0. The process has Markov property and is time-homogeneous.

The transition probabilities are determined as follows. Assume that Δ*t* can be chosen sufficiently small such that at most one change in state occurs during the time step Δ*t*. In particular, there can only be either a new colonization or decolonization on patients or HCWs, or a new unit of bacterial load (contamination) or a unit less of bacterial load (decontamination) in environment. The transition probabilities are written as follows:

4 $$\begin{array}{lll}{p}_{(s+{i}_{1},j+{i}_{2},k+{i}_{3});(s,j,k)}({\rm{\Delta }}t) & = & {\rm{Prob}}({\rm{\Delta }}{P}_{c},{\rm{\Delta }}{H}_{c},{\rm{\Delta }}{B}_{e})=({i}_{1},{i}_{2},{i}_{3})| ({P}_{c}(t),{H}_{c}(t),{B}_{e}(t))\\  & = & (s,j,k),\end{array}$$

where Δ*P*_*c*_ = *P*_*c*_(*t* + Δ*t*) − *P*_*c*_(*t*), and *i*_1_, *i*_2_, *i*_3_ ∈ {−1, 0, 1}. Hence,

5 $$\begin{array}{l}{p}_{(s+{i}_{1},j+{i}_{2},k+{j}_{3});(s,j,k)}({\rm{\Delta }}t)\\ \quad =\{\begin{array}{ll}\theta [{\gamma }_{u}({N}_{p}-s)+{\gamma }_{c}s]+{\alpha }_{p}{\beta }_{p}(1-\eta )({N}_{p}-s)j & \\ +\,{k}_{p}({N}_{h}-s)k{\rm{\Delta }}t, & ({i}_{1},{i}_{2},{i}_{3})=(1,0,0)\\ {\gamma }_{c}s{\rm{\Delta }}t, & ({i}_{1},{i}_{2},{i}_{3})=(-1,0,0)\\ \mbox{[}(1-\eta ){\alpha }_{p}{\beta }_{h}s({N}_{h}-j)+{k}_{h}({N}_{h}-j)k]{\rm{\Delta }}t, & ({i}_{1},{i}_{2},{i}_{3})=(0,1,0)\\ {\mu }_{c}j{\rm{\Delta }}t, & ({i}_{1},{i}_{2},{i}_{3})=(0,-1,0)\\ ({\nu }_{p}s+{\nu }_{h}j){\rm{\Delta }}t, & ({i}_{1},{i}_{2},{i}_{3})=(0,0,1)\\ {\gamma }_{b}k{\rm{\Delta }}t, & ({i}_{1},{i}_{2},{i}_{3})=(0,0,-1)\\ 1-\,\theta [{\gamma }_{u}({N}_{p}-s)+{\gamma }_{c}s]{\rm{\Delta }}t-{\alpha }_{p}{\beta }_{p}(1-\eta )({N}_{p}-s)j{\rm{\Delta }}t & \\ -\,{k}_{p}({N}_{h}-s)k{\rm{\Delta }}t-{\gamma }_{c}s{\rm{\Delta }}t & \\ -\,[(1-\eta ){\alpha }_{p}{\beta }_{h}s({N}_{h}-j)+{k}_{h}({N}_{j}-j)k]{\rm{\Delta }}t & \\ -\,{\mu }_{c}j{\rm{\Delta }}t-({\nu }_{p}s+{\nu }_{h}j){\rm{\Delta }}t-{\gamma }_{b}k{\rm{\Delta }}t, & ({i}_{1},{i}_{2},{i}_{3})=(0,0,0)\\ 0, & {\rm{otherwise}}.\end{array}\end{array}$$

We notice that the time step Δ*t* must be chosen to be sufficiently small such that all of these probabilities could stay in the interval [0, 1]. The transition matrix is too complicated to express, however, we could still write out the probabilities *p*_(*s*,*j*,*k*)_(*t* + Δ*t*) by using the Markov property:

6 $$\begin{array}{lll}{p}_{(s,j,k)}(t+{\rm{\Delta }}t) & = & {p}_{(s-1,j,k)}(t)\{\theta [{\gamma }_{u}({N}_{p}-s+1)+{\gamma }_{c}(s-1)]+{\alpha }_{p}{\beta }_{p}(1-\eta )\\  &  & \times \,({N}_{p}-s+1)j+{k}_{p}({N}_{p}-s+1)k\}{\rm{\Delta }}t+{p}_{(s+1,j,k)}(t){\gamma }_{c}(s+1){\rm{\Delta }}t\\  &  & +\,{p}_{(s,j-1,k)}(t)[(1-\eta ){\alpha }_{p}{\beta }_{h}s({N}_{h}-j+1)+{k}_{h}({N}_{h}-j+1)k]{\rm{\Delta }}t\\  &  & +\,{p}_{(s,j+1,k)}(t){\mu }_{c}(j+1){\rm{\Delta }}t+{p}_{(s,j,k-1)}(t)({\nu }_{p}s+{\nu }_{h}j){\rm{\Delta }}t\\  &  & +\,{p}_{(s,j,k+1)}(t){\gamma }_{b}(k+1){\rm{\Delta }}t+{p}_{(s,j,k)}(t)\{1-\{\theta {\gamma }_{u}({N}_{p}-s+1)\\  &  & +\,{\gamma }_{c}(s-1)+{\alpha }_{p}{\beta }_{p}(1-\eta )({N}_{p}-s)j+{\gamma }_{c}s\\  &  & +\,{k}_{p}({N}_{p}-s)k+(1-\eta ){\alpha }_{p}{\beta }_{h}s({N}_{h}-j)+{k}_{h}({N}_{h}-j)k\\  &  & +\,{\mu }_{c}j+{\nu }_{p}s+{\nu }_{h}j+{\gamma }_{b}k\}{\rm{\Delta }}t.\end{array}$$

Meanwhile, a system of forward Kolmogorov differential equations could be derived:

7 $$\begin{array}{lll}\frac{d{p}_{(s,j,k)}}{dt} & = & {p}_{(s-1,j,k)}\{\theta [{\gamma }_{u}({N}_{p}-s+1)+{\gamma }_{c}(s-1)]+{\alpha }_{p}{\beta }_{p}(1-\eta )({N}_{p}-s+1)j\\  &  & +\,{k}_{p}({N}_{p}-s+1)k\}+{p}_{(s+1,j,k)}{\gamma }_{c}(s+1)\\  &  & +\,{p}_{(s,j-1,k)}[(1-\eta ){\alpha }_{p}{\beta }_{h}s({N}_{h}-j+1)+{k}_{h}({N}_{h}-j+1)k]\\  &  & +\,{p}_{(s,j+1,k)}{\mu }_{c}(j+1)+{p}_{(s,j,k-1)}({\nu }_{p}s+{\nu }_{h}j)+{p}_{(s,j,k+1)}(t){\gamma }_{b}(k+1)\\  &  & +\,{p}_{(s,j,k)}\{1-\{\theta [{\gamma }_{u}({N}_{p}-s+1)+{\gamma }_{c}(s-1)]+{\alpha }_{p}{\beta }_{p}(1-\eta )\\  & \begin{array}{l}\end{array} & \times \,({N}_{p}-s)j+{k}_{p}({N}_{p}-s)k+{\gamma }_{c}s+(1-\eta ){\alpha }_{p}{\beta }_{h}s({N}_{h}-j)\\  &  & +\,{k}_{h}({N}_{h}-j)k+{\mu }_{c}j+{\nu }_{p}s+{\nu }_{h}j+{\gamma }_{b}k\}\}.\end{array}$$

## Results

### Numerical simulations of the deterministic model

**Figure 2:**
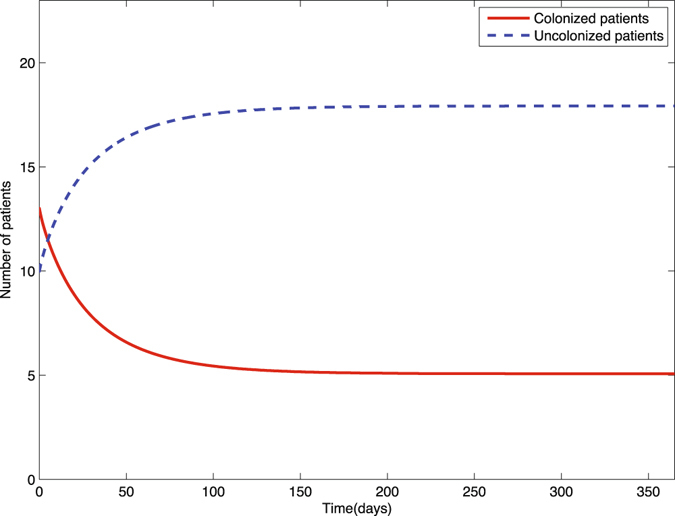
Solutions of colonized (*P*_*c*_(*t*)) and uncolonized (*P*_*u*_(*t*)) patients of the deterministic epidemic model (1) with initial values $({P}_{u}^{0},{P}_{c}^{0},{H}_{u}^{0},{H}_{c}^{0},{B}_{e}^{0})=(10,13,17,6,1000)$.

First we perform numerical simulations for solutions of the deterministic epidemic system. Once we obtain the estimates of parameters, it is the most efficient and direct way to check the result and its properties. We apply Euler’s method by using Matlab (Moehlis^38^). The stepsize is defined based on practical need. In general, smaller stepsizes will provide better simulations. However, it will increase the amount of calculation time of computer program. Thus, we have to choose an appropriate step size. Here, we define the step size *h* to be 0.01, and we have *R*_0_ = 0.7579 < 1. The initial values that we choose are $({P}_{u}^{0},{P}_{c}^{0},{H}_{u}^{0},{H}_{c}^{0},{B}_{e}^{0})=(10,13,17,6,1000)$. The numerical solutions of the deterministic epidemic model are given in Fig. 2.

**Figure 3:**
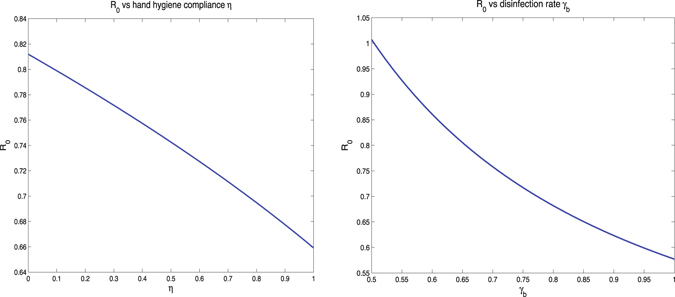
(**a**) *R*_0_ vs hand hygiene compliance *η*, and (**b**) *R*_0_ vs disinfection rate *γ*_*b*_.

**Figure 4:**
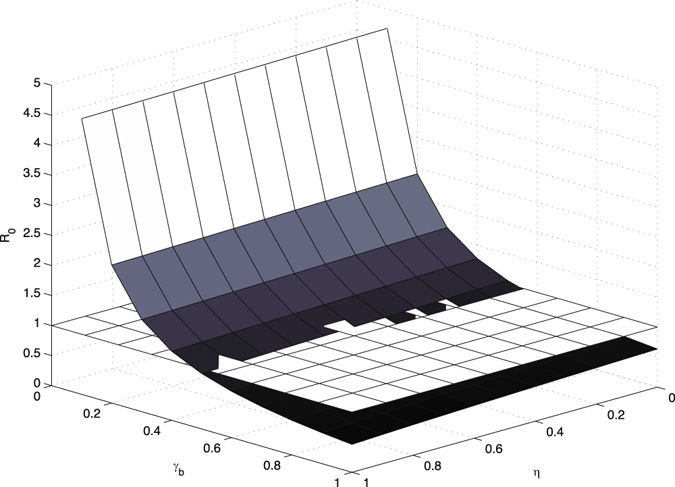
*R*_0_ vs hand hygiene compliance *η* and disinfection rate *γ*_*b*_, compared with the baseline plane of *R*_0_ = 1.

**Figure 5:**
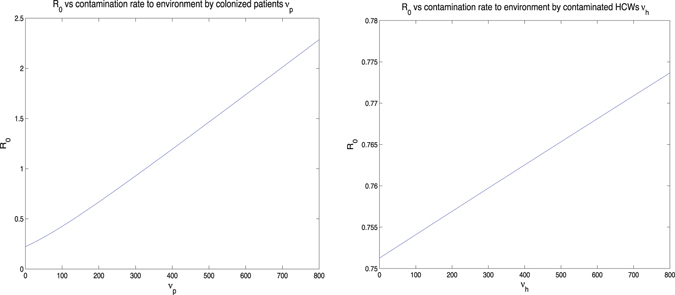
(**a**) *R*_0_ vs contamination rate of the environment by colonized patients *ν*_*p*_, and (**b**) *R*_0_ vs contamination rate of the environment by contaminated HCWs *ν*_*h*_.

**Figure 6:**
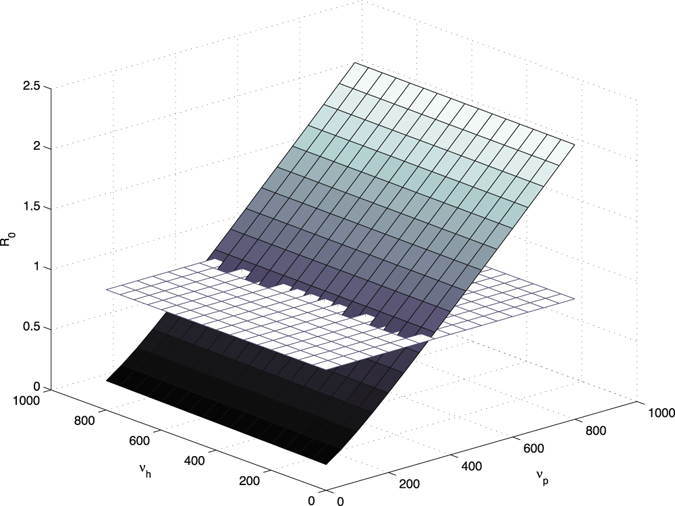
*R*_0_ vs contamination rate of the environment by colonized patients *ν*_*p*_ and contamination rate of the environment by contaminated HCWs *ν*_*h*_, compared with the baseline plane of *R*_0_ = 1.

**Figure 7:**
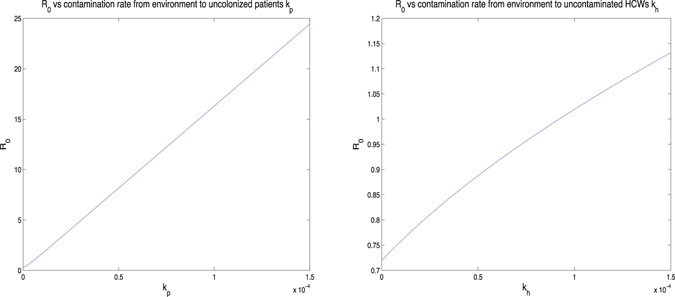
(**a**) *R*_0_ vs contamination rate from the environment to uncolonized patients *k*_*p*_, and (**b**) *R*_0_ vs contamination rate from the environment to uncontaminated HCWs *k*_*h*_.

**Figure 8:**
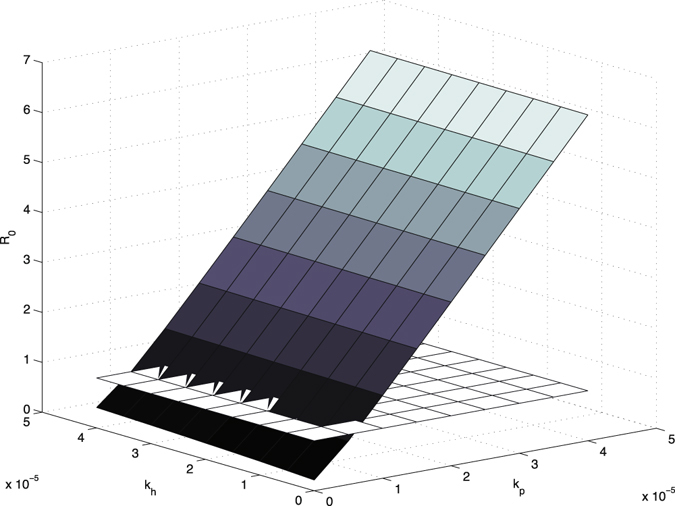
*R*_0_ vs colonization rate from the environment to uncolonized patients *k*_*p*_ and colonization rate from the environment to uncontaminated HCWs *k*_*h*_, compared with the baseline plane of *R*_0_ = 1.

In general, there are two types of analysis for determining how influential parameter variation affects the final model output: uncertainty analysis and sensitivity analysis. The uncertainty analysis is to determine the uncertainty in the model output, given the uncertainties in the parameter values. And sensitivity analysis means to quantitatively decide which parameters are most influential in the model output. In this paper, we focus on the sensitivity analysis, because two references (Wang *et al.* ^33^ and Wang *et al.* ^31^) have provided good resources for quantitative analysis. In this section, we will perform sensitivity analysis of *R*_0_ in terms of model parameters. We consider inputs by pairs. First, we consider the hand hygiene compliance and the disinfection rate of the environment. That is because that hand hygiene and disinfection of the hospital are both significantly important interventions. Figure 3 shows that if we increase the hand hygiene compliance of HCWs, *R*_0_ would be reduced substantially. Similarly, if we only consider to increase the disinfection rate of the environment, *R*_0_ would also be greatly decreased. Thus, it is necessary to check the output of combining these two control methods. The result has been shown in Fig. 4. When we increase both the hand hygiene compliance and disinfection rate, the basic reproduction number would drop dramatically. In numerical simulations, we assume that the contamination rate to environment of colonized patients *ν*_*p*_ is equal to the contamination rate to environment of contaminated HCWs *ν*_*h*_. However, in the sensitivity analysis, we will change them to observe their influences separately. Figure 5 gives a natural explanation of increasing contamination to the environment by colonized patients or contaminated HCWs. The basic reproduction number would increase in both cases. However, we notice that in Fig. 5(a), the increment of *R*_0_ is greater than that in Fig. 5(b), implying that it would be more effective to control the contamination rate to the environment by colonized patients than that of contaminated HCWs. Figure 6 presents the consequence of controlling both of them at the same time. Similarly, we consider the colonization rate from environment of uncolonized patients and uncontaminated HCWs under the same scalar in Fig. 7. It is easy to see that *R*_0_ would increase much more significantly when the contamination rate from environment to uncolonized patients increases, than that to uncontaminated HCWs. Combining with Fig. 5, it can be seen that it would be more important to control the contamination rate related to patients than to HCWs. Figure 8 shows the trend of *R*_0_ if we adjust both of them.

### Stochastic simulations

We first construct a stochastic epidemic model from the deterministic epidemic model (1), then use data in Table 1 to run stochastic simulations of our model.

The system has three variables with a joint probability

$${p}_{(s,j,k)}(t)={\rm{Prob}}\{{P}_{c}(t)=s,{H}_{c}(t)=j,{B}_{e}(t)=k\},$$

with *s* = 0, …, *N*_*p*_, *j* = 0, …, *N*_*h*_ and *k* ≥ 0, whose transition probabilities have been given in (5). Let *X*(*t*) = (*P*_*c*_(*t*), *H*_*c*_(*t*), *B*_*e*_(*t*))^*T*^, with infinitesimal Δ*X*(*t*) = (Δ*P*_*c*_(*t*), Δ*H*_*c*_(*t*), Δ*B*_*e*_(*t*))^*T*^. It is possible for us to write out the infinitesimal mean matrix *f*(*X*(*t*), *t*) as following:

8 $$E({\rm{\Delta }}X(t)| X(t))=(\begin{array}{c}{e}_{p}\\ {e}_{h}\\ {e}_{b}\end{array})\cdot {\rm{\Delta }}t=f(X(t),t)\cdot {\rm{\Delta }}t,$$

where

$$\begin{array}{lll}{e}_{p} & = & \theta [{\gamma }_{u}({N}_{h}-{P}_{c})+{\gamma }_{c}{P}_{c}]+{\alpha }_{p}{\beta }_{p}(1-\eta )({N}_{p}-{P}_{c}){H}_{c}+{k}_{p}({N}_{h}-{P}_{c}){B}_{e}-{\gamma }_{c}{P}_{c},\\ {e}_{h} & = & [(1-\eta ){\alpha }_{p}{\beta }_{h}{P}_{c}({N}_{h}-{H}_{c})+{k}_{h}({N}_{h}-{H}_{c}){B}_{e}]-{\mu }_{c}{H}_{c},\\ {e}_{b} & = & {\nu }_{p}{P}_{c}+{\nu }_{h}{H}_{c}-{\gamma }_{b}{B}_{e},\end{array}$$

and the infinitesimal variance matrix Σ(*X*(*t*), *t*) is given by:

9 $$E({\rm{\Delta }}X(t){({\rm{\Delta }}X(t))}^{T}| X(t))=(\begin{array}{ccc}{\delta }_{p} & 0 & 0\\ 0 & {\delta }_{h} & 0\\ 0 & 0 & {\delta }_{b}\end{array})\cdot {\rm{\Delta }}t=\sum (X(t),t)\cdot {\rm{\Delta }}t,$$

in which

$$\begin{array}{lll}{\delta }_{p} & = & \theta [{\gamma }_{u}({N}_{h}-{P}_{c})+{\gamma }_{c}{P}_{c}]+{\alpha }_{p}{\beta }_{p}(1-\eta )({N}_{p}-{P}_{c}){H}_{c}+{k}_{p}({N}_{h}-{P}_{c}){B}_{e}+{\gamma }_{c}{P}_{c},\\ {\delta }_{h} & = & [(1-\eta ){\alpha }_{p}{\beta }_{h}{P}_{c}({N}_{h}-{H}_{c})+{k}_{h}({N}_{h}-{H}_{c}){B}_{e}]+{\mu }_{c}{H}_{c},\\ {\delta }_{b} & = & {\nu }_{p}{P}_{c}+{\nu }_{h}{H}_{c}+{\gamma }_{b}{B}_{e}.\end{array}$$

It is easy to see that *δ*_*p*_, *δ*_*h*_, *δ*_*b*_ are all nonnegative. Diffusion matrix *G* is the solution of *GG* ^*T*^ = Σ. There may be several solutions of this equation depending on the expression of Σ, however, we could always pick the most visible one as

10 $$G=(\begin{array}{ccc}\sqrt{{\delta }_{p}} & 0 & 0\\ 0 & \sqrt{{\delta }_{h}} & 0\\ 0 & 0 & \sqrt{{\delta }_{b}}\end{array}).$$

Then the Itô SDE takes the following form:

11 $$dX(t)=f(X(t),t)dt+G(X(t),t)dW(t).$$

More precisely,

12 $$\left\{\begin{array}{ccc}\frac{d{P}_{c}(t)}{dt} & = & {e}_{p}dt+\sqrt{{\delta }_{p}}d{W}_{1}(t)\\ \frac{d{H}_{c}(t)}{dt} & = & {e}_{h}dt+\sqrt{{\delta }_{h}}d{W}_{2}(t)\\ \frac{d{B}_{e}(t)}{dt} & = & {e}_{b}dt+\sqrt{{\delta }_{b}}d{W}_{3}(t),\end{array}\right.$$

where *W*_1_, *W*_2_, *W*_3_ are three independent Wiener processes. If the terms associated with the Wiener processes are dropped, then we have the same ODE model as in (1).

Once we obtain the stochastic epidemic model, we are able to run stochastic simulations by using data in Table 1. Simulations are done by Matlab (Allen [ref. 37, chp. 3]). Consider variable *P*_*c*_ for a more specific explanation of this process. For *k* from 1 to *n*, where *n* is the path number of simulation; let *j* be the state from 1, then

13 $${P}_{c}(j+1,k)={P}_{c}(j,k)+{e}_{p}\cdot dt+\sqrt{{\delta }_{p}}\cdot \sqrt{dt}\cdot {r}_{p},$$

where *dt* = 0.01 is the time step, *r*_*p*_ is a standard normal random variable, and

$$\begin{array}{lll}{e}_{p} & = & \theta [{\gamma }_{u}\cdot \,{\rm{\max }}(({N}_{h}-{P}_{c}(j,k)),0)+{\gamma }_{c}{P}_{c}(j,k)]\\  &  & +\,{\alpha }_{p}{\beta }_{p}(1-\eta )\cdot \,{\rm{\max }}(({N}_{h}-{P}_{c}(j,k)),0)\cdot {H}_{c}(j,k)\\  &  & +\,{k}_{p}\cdot \,{\rm{\max }}(({N}_{h}-{P}_{c}(j,k)),0)\cdot {B}_{e}(j,k)-{\gamma }_{c}{P}_{c}(j,k),\\ {\delta }_{p} & = & \theta [{\gamma }_{u}\cdot \,{\rm{\max }}(({N}_{h}-{P}_{c}(j,k)),0)+{\gamma }_{c}{P}_{c}(j,k)]\\  &  & +\,{\alpha }_{p}{\beta }_{p}(1-\eta )\cdot \,{\rm{\max }}(({N}_{h}-{P}_{c}(j,k)),0)\cdot {H}_{c}(j,k)\\  &  & +\,{k}_{p}\cdot \,{\rm{\max }}(({N}_{h}-{P}_{c}(j,k)),0)\cdot {B}_{e}(j,k)-{\gamma }_{c}{P}_{c}(j,k).\end{array}$$

For each variable, we will present ten sample paths and compare them with the corresponding solution curves from the deterministic model. Usually, to verify whether a stochastic simulation is good or not, the mean and the variance of the difference between simulation and target value will be calculated. In this chapter, we will not check these means and variances due to the complexity of calculation. However, it is practicable to verify by observation: whether these sample paths are close to each other, and whether they have small noise according to the deterministic solution.

**Figure 9:**
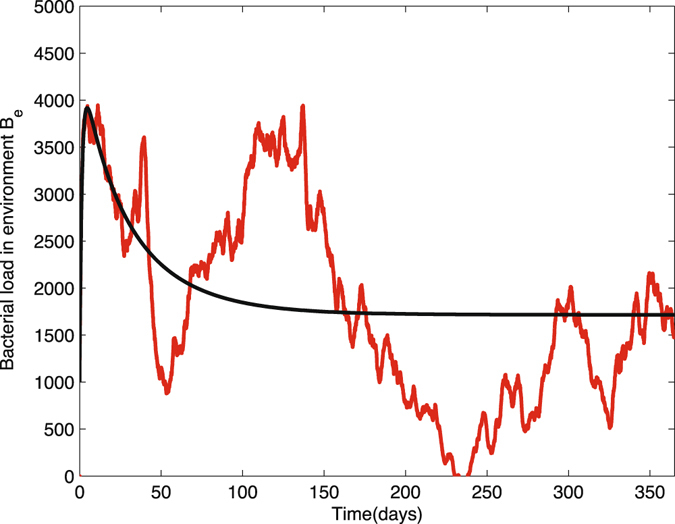
Ten sample paths of the bacterial load in environment in nosocomial infection model with environment infection are graphed with the deterministic solution (black curve). The parameter values are Δ*t* = 0.01, *N*_*p*_ = 23, *N*_*h*_ = 23, *θ* = 0.067, *α*_*p*_ = 0.0435, *β*_*p*_ = 0.72, *β*_*h*_ = 0.20, *η* = 0.4, *γ*_*u*_ = 0.067, *γ*_*c*_ = 0.046, *γ*_*b*_ = 0.7, *k*_*p*_ = 0.000004, *k*_*h*_ = 0.00001, (1 − *η*) = 0.6, *μ*_*c*_ = 24, *v*_*p*_ = 235, *v*_*h*_ = 235, time = 365, ${P}_{c}^{0}=13,{H}_{c}^{0}=6,{B}_{e}^{0}=1000$.

**Figure 10:**
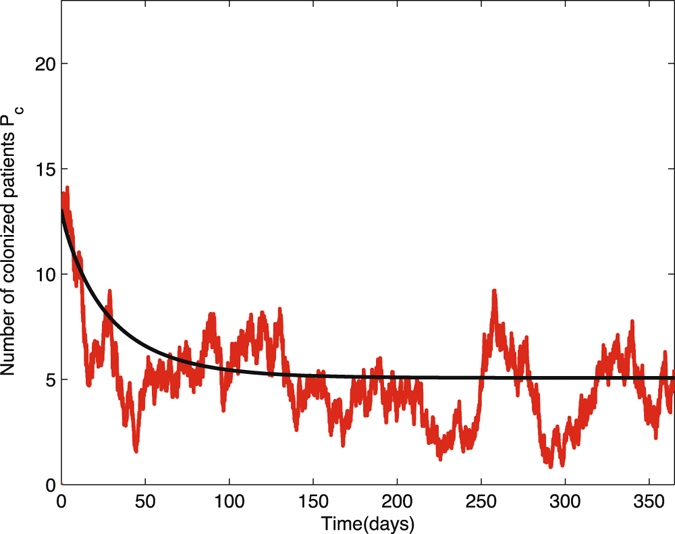
Ten sample paths of the number of colonized patients in nosocomial infection model with environment infection are graphed with the deterministic solution (black curve). The parameter values are Δ*t* = 0.01, *N*_*p*_ = 23, *N*_*h*_ = 23, *θ* = 0.067, *α*_*p*_ = 0.0435, *β*_*p*_ = 0.72, *β*_*h*_ = 0.20, *η* = 0.4, *γ*_*u*_ = 0.067, *γ*_*c*_ = 0.046, *γ*_*b*_ = 0.7, *k*_*p*_ = 0.000004, *k*_*h*_ = 0.00001, (1 − *η*) = 0.6, *μ*_*c*_ = 24, *v*_*p*_ = 235, *v*_*h*_ = 235, time = 365, ${P}_{c}^{0}=13,{H}_{c}^{0}=6,{B}_{e}^{0}=1000$.

**Figure 11:**
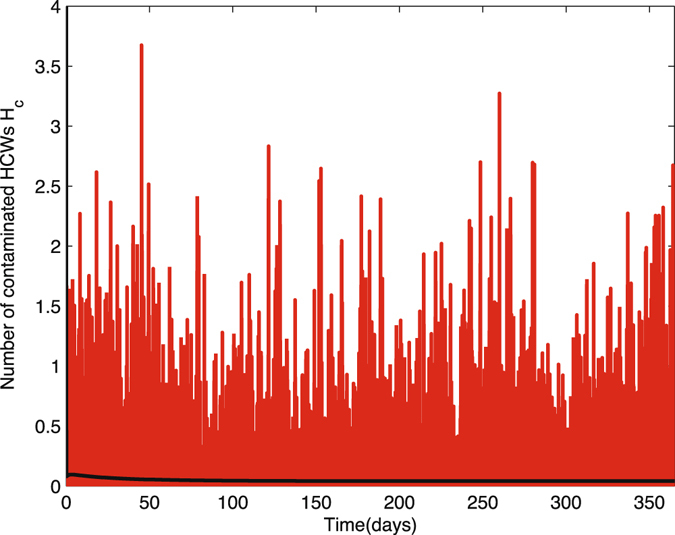
Ten sample paths of the number of contaminated HCWs in nosocomial infection model with environment infection are graphed with the deterministic solution (black curve). The parameter values are Δ*t* = 0.01, *N*_*p*_ = 23, *N*_*h*_ = 23, *θ* = 0.067, *α*_*p*_ = 0.0435, *β*_*p*_ = 0.72, *β*_*h*_ = 0.20, *η* = 0.4, *γ*_*u*_ = 0.067, *γ*_*c*_ = 0.046, *γ*_*b*_ = 0.7, *k*_*p*_ = 0.000004, *k*_*h*_ = 0.00001, (1 − *η*) = 0.6, *μ*_*c*_ = 24, *v*_*p*_ = 235, *v*_*h*_ = 235, time = 365, ${P}_{c}^{0}=13,{H}_{c}^{0}=6,{B}_{e}^{0}=1000$

We provide three figures as results of stochastic simulations on the bacterial load in the environment *B*_*e*_(*t*), the number of colonized patients *P*_*c*_(*t*), and the number of contaminated HCWs *H*_*c*_(*t*), compared with deterministic solution curves, respectively. As shown in Figs 9,10 and 11, in each run, ten sample paths are close to each other, and oscillate around the black solid curve, which is the solution curve of the deterministic system. Noticed that in the last figure, the deterministic solution curve is almost close to zero. This is because that the sizes of populations of patients and HCWs are both very small. However, the noise of this run was controlled to under four. Thus, we have provided a good explanation of the transmission dynamics for small populations with environment infection.

## Discussion

Traditional strategies of controlling nosocomial infection have been provided in many references (Wang *et al.* ^33^, Yong^39^). These measures include reducing the transmission rate between HCWs and patients, and the transmission rate between volunteers and patients; as well as raising the hand hygiene compliance of HCWs and volunteers. In this study, we introduced the environmental infection in a nosocomial infection model. Our research is devoted to suggesting more possibilities for determining the control intervention strategies, which are based on the sensitivity analysis of the basic reproduction number *R*_0_.

The first conclusion is that increasing the disinfection rate of environment will help to control the transmission dynamics of MRSA in the hospital. This includes 1) appropriate use of cleaners and disinfectants; 2) appropriate maintenance of medical equipment (e.g., automated endoscope reprocessors or hydrotherapy equipment); 3) adherence to water-quality standards for hemodialysis, and to ventilation standards for specialized care environments (e.g., airborne infection isolation rooms, protective environments, or operating rooms); and 4) prompt management of water intrusion into the facility (Sehulster and Chinn^40^). Meanwhile, it is essential to control the contamination rates between the environment and patients and HCWs. To be more specific, decreasing the contamination rate to the environment by colonized patients and contaminated HCWs, or decreasing the contamination rate from the environment to uncolonized patients and uncontaminated HCWs, will be helpful for controlling the infection in hospital. Two basic recommendations are limiting the scope of activities of patients, especially, for those high-risk patients, to avoid non-essential contacts with the environment, and increasing hand hygiene compliance of HCWs, in particular, before contacting any patient. It would be ideal if we could reduce both contamination rates to the environment from colonized patients and contaminated HCWs, and both contamination rates from the environment to uncolonized patients and uncontaminated HCWs. However, from the practical point of view, for instance, because of the financial budget and the lack of supervision, it is possible that not all of these transmission rates could be controlled at the same time. Our research indicates that under such situations, we should give priority to controlling the contamination rates related between the environment and patients. The sensitivity analysis of *ν*_*p*_ (i.e., contamination rate to environment from colonized patients) has explained that an increase of *ν*_*p*_ would increase the value of *R*_0_ dramatically, compared with the influence of the same increment of *ν*_*h*_ (i.e., contamination rate to environment from contaminated HCWs). Similarly, the sensitivity analysis of *k*_*p*_ (i.e., contamination rate from environment to uncolonized patients) has shown that under the same scale, the increase of *k*_*p*_ would result in huge jump of *R*_0_, compared with *k*_*h*_ (i.e., contamination rate from environment to uncontaminated HCWs). Thus, to reduce unnecessary contacts between patients and environment would decrease the transmission of MRSA significantly.

During the process of numerical simulations and sensitivity analysis, we apply data from the unit of EW (Emergency Ward) of Beijing Tongren Hospital from 3 March 2009 to 28 February 2010 (Wang *et al.* ^33^). There are both HCWs and volunteers working in the EW during the process of data collection. Since we do not consider the compartment of volunteers, it is not accurate to compare the patient data with solution of deterministic epidemic model or stochastic simulations. However, we still would able to estimate parameters from the original data.

In conclusion, decreasing the contamination rates between patients and environment, HCWs and environment, increasing the disinfection rate of environment, and increasing the hand hygiene compliance of HCWs would decrease MRSA transmission remarkably.

## Electronic supplementary material

Supplementary Materials
