# Supplementary material for: Modeling Nosocomial Infections of Methicillin-Resistant Staphylococcus aureus with Environment Contamination*
Source: Sci Rep. 2017 Apr 3;7:580. doi: 10.1038/s41598-017-00261-1 (PMC5428062; doi:10.1038/s41598-017-00261-1)
Supplement: Supplementary file 1 — Supplementary Materials [file 41598_2017_261_MOESM1_ESM.pdf]

Supplementary Material

Modeling Nosocomial Infections of Methicillin-Resistant *Staphylococcus aureus* with Environment Contamination

Lei Wang and Shigui Ruan

We derive R_0 by using the definition notations and technique of Diekmann et al. [1] and van den Driessche and Watmough [3]. When $\theta = 0$, that is no colonized patients are admitted into hospital, the disease-free equilibrium (DFE) is defined to be

$$E_0 = (P_u, P_c, H_u, H_c, B_e) = (N_p, 0, N_h, 0, 0),$$

where N_p, N_h are total number of patients and HCWs, respectively. The infected compartments are colonized patients P_c, contaminated HCWs H_c and bacterial load B_e; the uninfected compartments are uncolonized patients P_u and uncontaminaed HCWs H_u. Thus, for our model, $n = 5, m = 3$. After rearrangement, we denote

$$x = (P_c, H_c, B_e, P_u, H_u)^T, \quad x_0 = (0, 0, 0, N_p, N_h),$$

and

$$\dot{x_i} = f_i(x) = \mathcal{F}_i(x) - (\mathcal{V}_i(x)),$$

with

$$\mathcal{F}(x) = \begin{pmatrix} \mathcal{F}_1(x) \\ \mathcal{F}_2(x) \\ \mathcal{F}_3(x) \\ \mathcal{F}_4(x) \\ \mathcal{F}_5(x) \end{pmatrix} = \begin{pmatrix} \alpha_p\beta_p(1-\eta)P_uH_c + k_pP_uB_e \\ (1-\eta)\alpha_p\beta_hP_cH_u + k_hH_uB_e \\ 0 \\ 0 \\ 0 \end{pmatrix}, \tag{S1}$$

and

$$\mathcal{V}(x) = \begin{pmatrix} \mathcal{V}_1(x) \\ \mathcal{V}_2(x) \\ \mathcal{V}_3(x) \\ \mathcal{V}_4(x) \\ \mathcal{V}_5(x) \end{pmatrix} = \begin{pmatrix} \gamma_cP_c - \theta(\gamma_uP_u + \gamma_cP_c) \\ \mu_cH_c \\ \gamma_bB_e - (\nu_pP_c + \nu_hH_c) \\ \alpha_p\beta_p(1-\eta)P_uH_c + k_pP_uB_e + \gamma_uP_u - (\gamma_uP_u + \gamma_cP_c) \\ (1-\eta)\alpha_p\beta_hP_cH_u + k_hH_uB_e - \mu_cH_c \end{pmatrix}, \tag{S2}$$

It is easy to check that the assumptions in van den Driessche and Watmough [3] are satisfied. Thus,

$$\begin{aligned} F &= \left[\frac{\partial \mathcal{F}_i}{\partial x_j}(x_0)\right] \\ &= \begin{pmatrix} -\alpha_p\beta_p(1-\eta)H_c - k_pB_e & \alpha_p\beta_p(1-\eta)(N_p - P_c) & k_p(N_p - P_c) \\ (1-\eta)\alpha_p\beta_h(N_h - H_c) & -(1-\eta)\alpha_p\beta_hP_c - k_hB_e & k_h(N_h - H_c) \\ 0 & 0 & 0 \end{pmatrix}\Bigg|_{x_0} \\ &= \begin{pmatrix} 0 & \alpha_p\beta_p(1-\eta)N_p & k_pN_p \\ (1-\eta)\alpha_p\beta_hN_h & 0 & k_hN_h \\ 0 & 0 & 0 \end{pmatrix}, \end{aligned} \tag{S3}$$

and

$$V = \left[\frac{\partial \mathcal{V}_i}{\partial x_j}(x_0)\right] = \begin{pmatrix} \theta\gamma_u + (1-\theta)\gamma_c & 0 & 0 \\ 0 & \mu_c & 0 \\ -\nu_p & -\nu_h & \gamma_b \end{pmatrix}, \tag{S4}$$

then

$$V^{-1} = \frac{1}{\gamma\mu_c\gamma_b}\begin{pmatrix} \mu_c\gamma_b & 0 & 0 \\ 0 & \gamma\gamma_b & 0 \\ \nu_p\mu_c & \gamma\nu_h & \mu_c\gamma \end{pmatrix}, \tag{S5}$$

with $\gamma = \theta\gamma_u + (1-\theta)\gamma_c$.

Hence,

$$\begin{aligned} FV^{-1} &= \frac{1}{\gamma\mu_c\gamma_b}\begin{pmatrix} 0 & \alpha_p\beta_p(1-\eta)N_p & k_pN_p \\ (1-\eta)\alpha_p\beta_hN_h & 0 & k_hN_h \\ 0 & 0 & 0 \end{pmatrix} \cdot \begin{pmatrix} \mu_c\gamma_b & 0 & 0 \\ 0 & \gamma\gamma_b & 0 \\ \nu_p\mu_c & \gamma\nu_h & \gamma\mu_c \end{pmatrix} \\ &= \frac{1}{\gamma\mu_c\gamma_b}\begin{pmatrix} k_p\nu_p\mu_cN_p & [\alpha_p\beta_p(1-\eta)\gamma_b + k_p\nu_h]\,\gamma N_p & k_p\gamma\mu_cN_p \\ ((1-\eta)\alpha_p\beta_h\gamma_b + k_h\nu_p)\mu_cN_h & k_h\gamma\nu_hN_h & k_h\gamma\mu_cN_h \\ 0 & 0 & 0 \end{pmatrix}. \end{aligned} \tag{S6}$$

The basic reproduction number is defined the spectral radius of FV^{-1}:

$$\begin{aligned} R_0 &= \frac{k_p\nu_pN_p}{2\gamma\gamma_b} + \frac{k_h\nu_hN_h}{2\mu_c\gamma_b} \\ &\quad + \frac{\sqrt{(k_p\nu_p\mu_cN_p - k_h\nu_h\gamma N_h)^2 + 4[(\alpha_p\beta_p(1-\eta)\gamma_b + k_p\nu_h)\,((1-\eta)\alpha_p\beta_h\gamma_b + k_h\nu_p)\mu_c\gamma N_hN_p]}}{2\gamma\mu_c\gamma_b}. \end{aligned}$$

One can see that if $P_u^0, P_c^0, H_u^0, H_c^0, B_e^0 \geqslant 0$, then solutions are non-negative and remain bounded in the positively invariant set in $\mathbb{R}^5$

$$G := \{(P_u, P_c, H_u, H_c, B_e) \in \mathbb{R}_+^5 : P_u + P_c + H_u + H_c + B_e \leqslant N\},$$

where N is a fixed integer.

In fact, it is easy to see that the solutions remain in the positive cone if the initial conditions are in the positive cone (Smith and Waltman [2, App. B]). Let $T(t) = P_u(t) + P_c(t) + H_u(t) + H_c(t) + B_e(t)$. From (**??**) we have

$$\begin{aligned} \frac{dT(t)}{dt} = \frac{dB_e(t)}{dt} &= \nu_pP_c(t) + \nu_hH_c(t) - \gamma_bB_e(t) \\ &\leqslant \nu_pN_p + \nu_hN_h - \gamma_bB_e(t), \end{aligned}$$

which implies that

$$B_e(t) \leqslant \frac{(\nu_pN_p + \nu_hN_h)}{\gamma_b}(1 - e^{-\gamma_bt}) + B_e^0e^{-\gamma_bt}.$$

So $B_e(t)$ is bounded by a fixed number

$$M = \frac{(\nu_pN_p + \nu_hN_h)}{\gamma_b} + B_e^0.$$

Let $N = N_p + N_h + M$, then

$$P_u(t) + P_c(t) + H_u(t) + H_c(t) + B_e(t) \leqslant N.$$

Thus, the solutions remain bounded in a positive cone of $\mathbb{R}^5$, and the system induces a global semiflow in the positively invariant set G of $\mathbb{R}^5$.

When $\theta = 0$, following a result of van den Driessche and Watmough [3], we know that if $R_0 < 1$, the disease-free steady state $(N_p, 0, N_h, 0, 0)$ is locally asymptotically stable; if $R_0 > 1$, the disease-free steady state is unstable.

References

[1] Diekmann, O., Heesterbeek, J.A.P. & Roberts, M.G. The construction of next-generation matrices for compartmental epidemic models, *J. Royal Soc. Interface* **7**, 873-885 (2010).

[2] Smith, H. L. & Waltman, P. E. *The Theory of the Chemostat: Dynamics of Microbial Competition,* (Cambridge University Press, Cambridge, 1995).

[3] van den Driessche, P. & Watmough, J. Reproduction numbers and sub-threshold endemic equilibria for compartmental models of disease transmission, *Math. Biosci.* **180**, 29-48 (2002).
